# Distinct differences of vertical phytoplankton community structure in mainstream and a tributary bay of the Three Gorges Reservoir, China

**DOI:** 10.3389/fpls.2024.1381798

**Published:** 2024-03-22

**Authors:** Lan Wang, Lu Tan, Qinghua Cai

**Affiliations:** ^1^ State Key Laboratory of Freshwater Ecology and Biotechnology, Institute of Hydrobiology, Chinese Academy of Sciences, Wuhan, Hubei, China; ^2^ Hubei Key Laboratory of Wetland Evolution & Ecological Restoration, Wuhan Botanical Garden, Chinese Academy of Sciences, Wuhan, Hubei, China

**Keywords:** vertical distribution, phytoplankton community, β diversity, water level, stratification

## Abstract

The vertical distribution of phytoplankton plays a crucial role in shaping the dynamics and structure of aquatic communities. In highly dynamic reservoir systems, water level fluctuations significantly affect the physiochemical conditions and the phytoplankton community. However, the specific effects on the vertical characteristics of phytoplankton between the mainstream and the tributary bay of the reservoir remain unstudied. This study investigated the vertical aspects of phytoplankton density, biomass, α and β diversity through monthly sampling over two years in the mainstream (Chang Jiang, CJ) and a tributary bay (Xiang Xi, XX) of the Three Gorges Reservoir in China. Phytoplankton density and biomass were significantly higher in XX, indicating an increased risk of algal blooms in the tributary. The phytoplankton community in CJ showed more stable species-environment relationships, a lower Shannon index and a higher evenness index, suggesting a relatively simple structure and a more uniform distribution of phytoplankton among different water layers. Conversely, XX showed greater differences between water layers (higher β diversity), with significant negative correlations with water level and positive correlations with DO difference, dissolved silica (DSi) difference, and stratification. Peak phytoplankton density and biomass, as well as high β diversity in XX, occurred during periods of decreased water levels with strong stratification in spring and summer. A structural equation model complemented by path analysis revealed that a decrease in water level could increase β diversity either directly through internal processes with extended residence time or indirectly by modifying stratification and the vertical distribution of DSi in XX. Therefore, a proposed water quality management strategy for XX was to increase the water level or reduce β diversity by implementing artificial mixing during stratification periods. Overall, this study lies in its comprehensive investigation of the vertical characteristics of the phytoplankton community in both the mainstream and the tributary bay of the Three Gorges Reservoir, elucidating the significant impact of water level fluctuations and providing insights for targeted water quality management strategies in the tributary bay to mitigate potential ecological impacts.

## 1 Introduction

River systems worldwide are increasingly affected by damming to meet growing demands for hydropower, flood control, and reliable water resources (Straškraba, 2005). The impoundment of reservoirs significantly alters river flows, shorelines, sediments, and aquatic organisms. These effects are particularly complex and profound in the tributaries of reservoirs, where hydrodynamics and water mass transport processes are significantly altered. Consider the Three Gorges Project, the world’s largest hydraulic engineering project. With the implementation of the Three Gorges Dam Project in 2008, the reservoir water level fluctuated from 145 m above sea level in summer to 175 m above sea level in winter (first recorded on October 26, 2010). This resulted in a water level fluctuation zone of about 350 km^2^ in the reservoir (New and Xie, 2008). Before the impoundment, the tributaries maintained a natural river state, with the water environment mainly determined by the characteristics of the tributary basin. However, after impoundment, the rising water level in the mainstream induces a flow back into the tributary (Liu et al., 2012). This results in a slowing of the flow velocity, a reduction in the aquatic environment capacity, and an influx of additional nutrients from the mainstream, further degrading the aquatic environment. As a result, the likelihood of algal blooms is significantly increased (Wang et al., 2011; Liu et al., 2012; Li et al., 2021).

Phytoplankton is widely recognized as the primary producer in aquatic ecosystems and plays a crucial ecological role. However, their abnormal proliferation, known as algal blooms, has negative impacts on the aquatic environment. Their sensitivity to environmental changes underscores their importance in understanding and mitigating environmental challenges. Therefore, it serves as a good indicator of water quality and ecosystem health (Phillips et al., 2013; Bellinger and Sigee, 2015). The vertical distribution of phytoplankton is fundamental to the dynamics and structure of aquatic communities (Ryabov et al., 2010). Increased mixing has been identified as a key strategy to mitigate algal blooms (Visser et al., 2016; Wen et al., 2022), and increasing mixing promotes uniformity in the vertical distribution of phytoplankton communities across different layers in terms of density, biomass, and compositional structure. This uniformity in the vertical distribution of phytoplankton has emerged as a reliable indicator of the effectiveness of water quality regulation under hydrodynamic conditions. Most related studies have traditionally focused on reporting vertical phytoplankton profiles based on total density (Ryabov et al., 2010; Ikram et al., 2017; Chen et al., 2021; Huang et al., 2022), total biomass (Ji et al., 2017; Xue et al., 2017; Sakharova and Korneva, 2018; Cordero-Bailey et al., 2021; Lu et al., 2023; Radchenko et al., 2023), or species composition (Ryabov et al., 2010; Mojica et al., 2015; Ikram et al., 2017; Sakharova and Korneva, 2018; Chen et al., 2021; Huang et al., 2022; Cui et al., 2023; Lu et al., 2023; Radchenko et al., 2023), with limited attention to diversity (Sakharova and Korneva, 2018; Cui et al., 2023). Diversity is also an important community attribute, and various reviews have concluded that diversity plays a major role in maintaining ecosystem productivity and stability (Cardinale et al., 2012; Tilman et al., 2014). Therefore, a comprehensive study of the vertical distribution patterns of phytoplankton in reservoir ecosystems serves as a valuable complement to current research and has practical implications for the development of effective human-driven regulation strategies in reservoirs.

The formation of vertical phytoplankton profiles is influenced by several primary factors. One factor is light intensity, which decreases with depth, making deep layers unfavorable for photosynthetic phytoplankton species. Another factor is the nutrient gradient, which supports diverse production for phytoplankton with different nutrient requirements. In addition, different water temperatures at different depths in a reservoir can directly affect phytoplankton, and the stratification of water quality conditions, along with changes in relative water column stability caused by thermal stratification, can indirectly affect phytoplankton dynamics (Cui et al., 2021). In many ecosystems, mixing and stratification play an essential role as key drivers of the distribution and segregation of different phytoplankton taxa (Mojica et al., 2015). In artificially regulated reservoirs, all of these factors can be influenced by hydrodynamic factors such as water level. Numerous studies have highlighted the potential to mitigate algal blooms by regulating hydrodynamic conditions such as water level fluctuations (Bakker and Hilt, 2015; Yang et al., 2016; Ji et al., 2017). The effect of hydrological factors on phytoplankton has become a research hotspot in recent years, as it is not only a key factor influencing phytoplankton growth, but also the most easily regulated factor in reservoirs (Cui et al., 2021). The effect of water level on phytoplankton biomass and composition in reservoir systems has been well studied (Naselli-Flores and Barone, 1997; Mac Donagh et al., 2009; Wang et al., 2011; Zhu et al., 2013; Yang et al., 2016). However, its influence on the vertical structure of phytoplankton and the underlying mechanisms are still lacking.

This study, conducted over two annual cycles with monthly sampling, examines the vertical distribution patterns of phytoplankton under the influence of different environmental factors and water levels. The primary objective is to explore potential differences in the vertical distribution of phytoplankton between the mainstream and the tributary of the Three Gorges Reservoir, and to understand how these differences variances respond to changes in hydrodynamic and environmental conditions. We hypothesized that the water level had both direct and indirect effects on the phytoplankton community and addressed the potential pathways of influence. The results will provide valuable scientific information for targeted management and conservation of the ecological systems within the mainstream and tributary of the Three Gorges Reservoir.

## 2 Materials and methods

### 2.1 Study site and sampling

**Figure 1:**
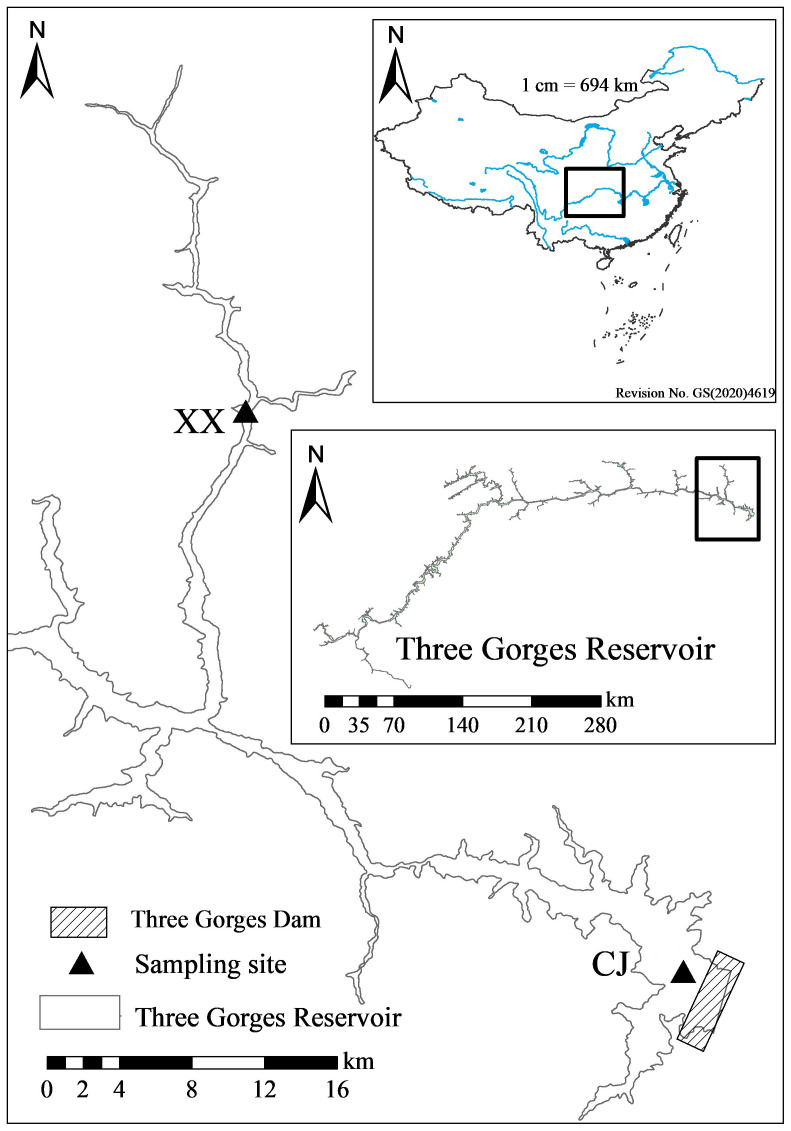
Map of sampling sites in this study.

The Three Gorges Reservoir (TGR) is located at 29°16’-31°25’ N, 106°-110°50’ E (Huang et al., 2006), with a subtropical monsoon climate prevails (Jiang et al., 2006). It has an average annual precipitation of 1,000-1,300 mm, characterized by warm winter and hot summer, early spring and cold autumn (Cai et al., 2010). It is more than 600 km long and 1.1 km wide, with a surface area of 1,080 km^2^ (Huang et al., 2006). The sampling site for the mainstream of TGR (Chang Jiang, CJ) is located near the dam, 31 km from the mouth of the Xiangxi River (**Figure 1**). The Xiangxi River is the largest tributary of the TGR in Hubei Province, with a mainstream length of 94 km and a watershed area of 3,099 km^2^ (Wang et al., 1997). The lower 20-40 km section of Xiangxi River was named Xiangxi Bay after the impoundment of the TGR (Cai and Hu, 2006). The sampling site for the tributary of the TGR (Xiang Xi, XX) was located near the middle of Xiangxi Bay, 18 km from the mouth of the Xiangxi River (**Figure 1**). The CJ and XX sites have been sampled as a representative of the mainstream and tributary of the TGR since the early study by Kuang et al. (2005).

Sampling was performed monthly from September 2008 through August 2010. A 5 L Van Dorn sampler was used to collect water samples at depths of 0.5 m, 2 m, 5 m, 10 m, 15 m, and 20 m for both CJ and XX. 1 L samples for phytoplankton analysis were fixed with neutral Lugol’s solution immediately after sampling. 250 ml samples for nutrient analysis were stored in a pre-cleaned plastic bottle and acidified with sulfuric acid for laboratory analysis.

### 2.2 Biotic and abiotic variable measurements

Phytoplankton was quantitatively analyzed in a Fuchs-Rosental slide using an Olympus CX21 microscope (Olympus Corporation, Japan) at 400× magnification. Previously, a sedimentation method was used to concentrate the phytoplankton (Huang et al., 2000; Cai, 2007). Taxonomic identification was performed according to the guidelines of Hu and Wei (2006) and John et al. (2002). Vertical profiles of water temperature (Temp, °C), conductivity (Cond, ms/cm), dissolved oxygen (DO, mg/L), pH, and turbidity (Turb, NTU) were measured using environmental monitoring systems (YSI 6600EDS, USA). Concentrations of total nitrogen (TN), nitrate-nitrogen (NO_3_-N), total phosphorus (TP), phosphate-phosphorus (PO_4_-P), and dissolved silicon (DSi) were measured with a segmented flow analyzer (Skalar San^++^, Netherlands) according to the Protocols for Standard Observation and Measurement in Aquatic Ecosystems of the Chinese Ecosystem Research Network (CERN) (Huang et al., 2000; Cai, 2007).

### 2.3 Data analysis

Algal biovolume was calculated using formulas for geometric shapes, with the fresh weight unit expressed in mass, where 1 mm^3^/L is equal to 1 mg/L (Huang et al., 2000; Wetzel and Likens, 2000). Real-time data on water level, inflow, and outflow data for the TGR were obtained from the China Three Gorges Corporation. Linear correlation between environmental variables was assessed using Pearson’s correlation coefficient. To examine the relationship between phytoplankton community (density, biomass, and α diversity index Shannon and evenness) and environmental variables, we performed a Mantel test using the R package “LinkET”.

Constrained ordination methods including Redundancy Analysis (RDA) and Canonical Correspondence Analysis (CCA), are used to examine the relationships between phytoplankton community structure and environmental factors. The decision to use either RDA (maximum DCA axis length below 3) or CCA (maximum DCA axis length above 4) ordination methods is based on Detrended Correspondence Analysis (DCA) applied to the species data, according to (Lepš and Šmilauer, 2003). In the RDA and CCA analysis, the significant environmental variables are identified using the “envfit” function.

The COSTATIS technique, which is based on Partial Triadic Analysis combined with Co-Inertia Analysis, was used to assess the stability of phytoplankton species-environment relationships using the R packages “ade4” and “adegraphics” (Slimani et al., 2017). COSTATIS brings to light the connections between two stable structures (a set of species data tables and a set of environmental parameter tables). The use of COSTATIS eliminates conflicting variation between the entire sequences are eliminated, thereby facilitating ease of interpretation (Thioulouse et al., 2004).

The Shannon index (Shannon) and Buzas and Gibson’s evenness index (Evenness) were chosen to characterize α diversity. The Shannon index takes into account the number of individuals as well as the number of taxa and is calculated using the formula:


$$\text{Shannon }=\;-\text{sum}\left(\left({\text{n}}_{\text{i}}/\text{n}\right)\text{ln}\left({\text{n}}_{\text{i}}/\text{n}\right)\right)$$

where n_i_ is the number of individuals of taxon i and n is the total number of individuals. The Buzas and Gibson’s evenness index is calculated as:


$$\mathrm{Evenness}\;=\;{\text{e}}^{\text{Shannon}}/\text{S}$$

where S is the number of taxa. The Friedman test was used to compare physical and chemical conditions, total density and biomass, and Shannon and evenness among different water layers for both CJ and XX. The Kruskal-Wallis H test was used to compare the physical and chemical conditions, total density and biomass, and Shannon and evenness between CJ and XX. Bray-Curtis dissimilarity was used to measure the β diversity among samples based on phytoplankton community, while Euclidean distance was used to assess the dissimilarity among samples with respect to environmental factors. Mantel test was performed to select significant environmental factors related to Bray-Curtis dissimilarity of the community. All the DCA, RDA, CCA, Shannon and evenness index, β diversity and Mantel test were calculated using the “vegan” package in R.

Two hydrodynamic factors were examined for their influence on the α and β diversity of the phytoplankton community: squared buoyancy frequency (N^2^) and water level. Squared buoyancy frequency (N^2^) can serve as an alternative indicator of stratification (Li et al., 2020) and is calculated using the formula:


$${\text{N}}^{2}=\left(\text{g}/{\text{ρ}}_{0}\right)/\left(\text{dρ}/\text{dz}\right)$$

Where g is the gravitational acceleration (9.8092597 m/s^2^), ρ_0_ is the density of water at 3.98°C, and z is the water depth. In our study, N^2^ is calculated for the entire water column, extending from the surface to a depth of 20 m, to assess the degree of stratification of the entire water column. A higher N^2^ indicates a more stratified water column and a higher static stability. Eleven elementary indices were used to clarify the water level in three aspects: magnitude, frequency and rate of change of water level data, following the approach proposed by (Olden and Poff, 2003). These indices were calculated on the day of sampling and 1 to 30 days prior to sampling, with the aim of assessing the temporal effects of water level fluctuations on α and β diversity. The calculation of these indices was performed using the “SER” function within the R package “SER” (Guo et al., 2020). The univariate linear regression model was used to investigate the relationships between the β diversity and the Euclidean distance of environmental factors, as well as a single key environmental factor determined by the Mantel test. It was also used to investigate the response of α and β diversity to N^2^ and water level over different time scales. In addition, a structural equation model (SEM) with path analysis was used to test the direct and indirect effects of the predictor variables (Shipley, 2002). Significant paths were retained while non-significant paths were removed from the developed model. The final model was validated using the Chi-square *p-*value, the goodness-of-fit index (GFI), the adjusted goodness-of-fit index (AGFI), and the root mean square error of approximation (RMSEA). Structural equation analysis was performed using the “lavaan” package in R.

## 3 Results

### 3.1 Hydrodynamic and environmental conditions

**Figure 2:**
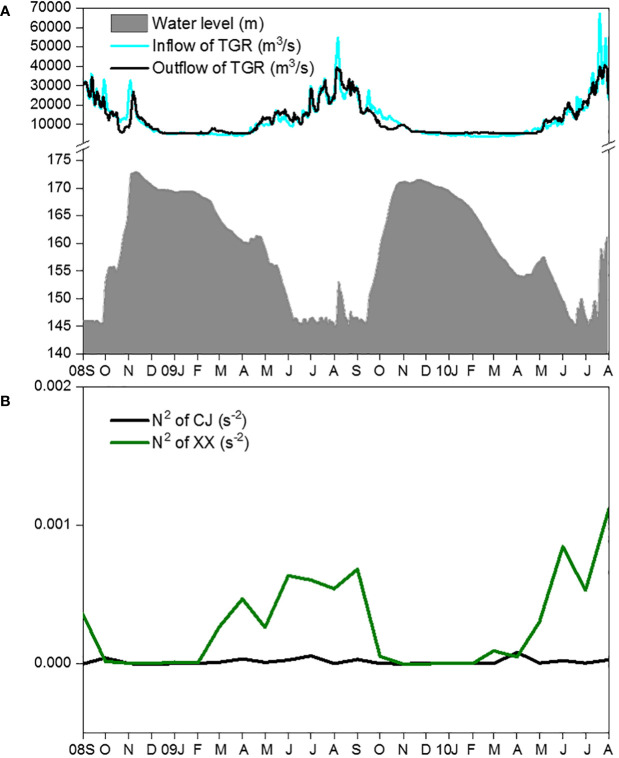
Changes of water level, inflow and outflow of TGR **(A)** and vertical stratification **(B)** during the study period.

The main hydrodynamic conditions, including water level (**Figure 2A**), inflow and outflow of the TGR (**Figure 2B**), showed regular intra-annual variations during the two-year study period. The mean daily water level showed higher values from November to the following January and remained low from June to September. TGR inflow and outflow peaked from July to September and remained low from late November to the following March. From February to September, N^2^ values in XX were significantly higher than those in CJ (**Figure 2B**). However, from October to the following January, the water column in both CJ and XX showed little stratification (indicating strong mixing).

**Figure 3:**
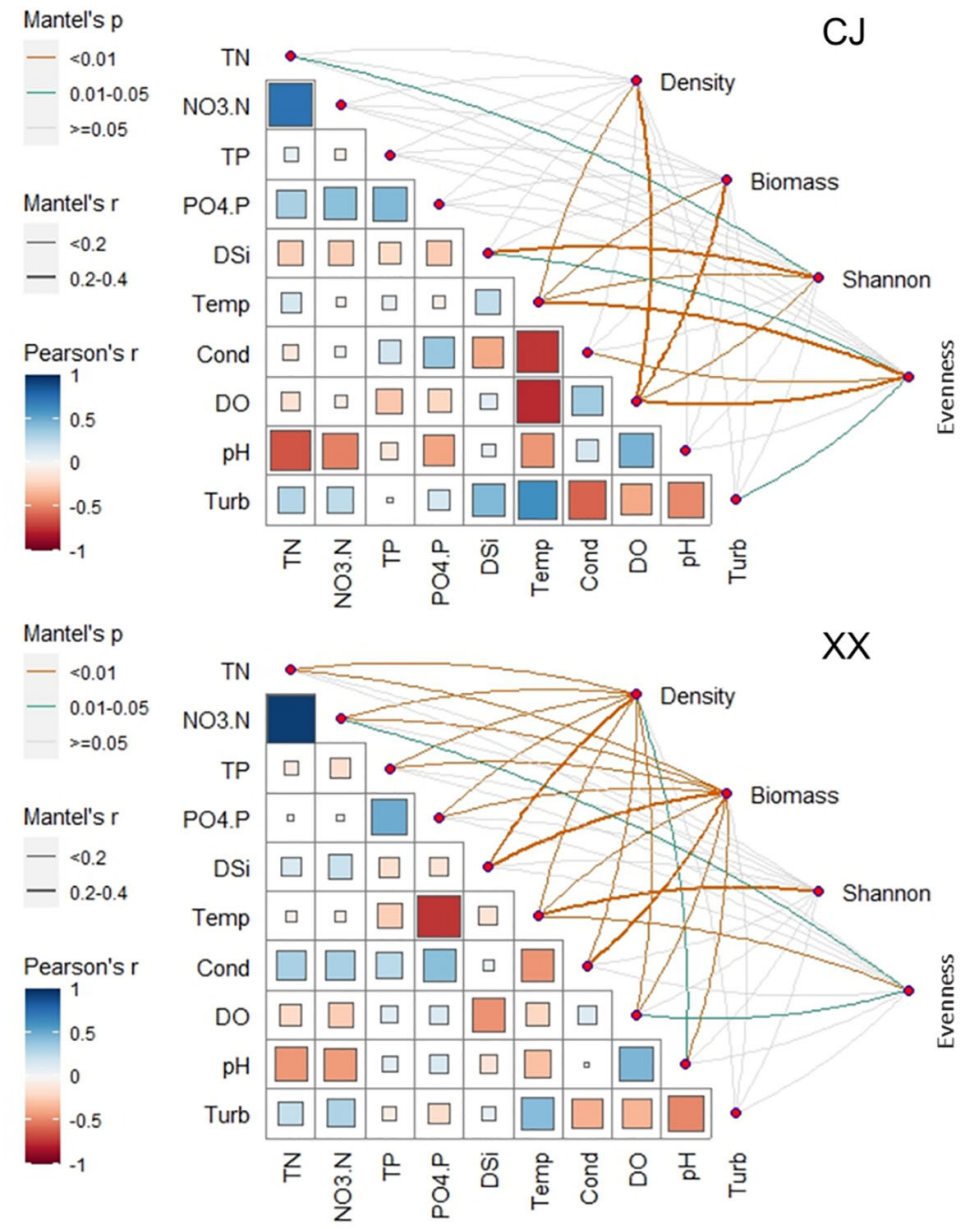
Relationship between environmental variables and phytoplankotn density, biomass, α diversity indices (Shannon and Evenness) through Mantel tests. Pairwise comparisons of different enviornmental variables are presented in the bottom-left section.

Except for water temperature, pH and turbidity, significant differences were observed in most environmental conditions between CJ and XX (**Table 1**). Among water layers in CJ, TN, NO_3_-N, PO_4_-P, DSi and Turb showed no significant differences, while other environmental factors showed remarkable differences. In XX, most environmental factors showed significant differences among different water layers, except for Turb. TN, NO_3_-N, TP, PO_4_-P, DSi, and conductivity were significantly higher in CJ compared to XX, while XX showed significantly higher DO. The correlation analysis between environmental factors in **Figure 3** showed that in both CJ and XX, TN and NO_3_-N had relatively high positive correlations. In CJ, relatively high negative correlations were observed between Temp and Cond, and between Temp and DO. In XX, a relatively high negative correlation was observed between Temp and PO_4_-P.

**Table 1:** Statistical summary of environmental conditions, phytoplankton density, biomass and diversity for CJ and XX.

	CJ			XX			Differences between CJ and XX
	Mean	Min	Max	Mean	Min	Max	
TN (mg/L)	1.73	0.94	2.54	1.38^a^	0.56	2.54	***
NO_3_-N (mg/L)	1.49	0.85	2.11	1.21^c^	0.45	1.98	***
TP (mg/L)	0.115^a^	0.02	0.73	0.093^c^	0.01	0.55	***
PO_4_-P (mg/L)	0.085	0.01	0.26	0.064^c^	0.01	0.15	***
DSi (mg/L)	3.62	2.5	7.1	3.02^c^	0.3	6.1	**
Temp (°C)	18.67^c^	10.76	25.47	19.22^c^	10.27	30.71	
Cond (ms/cm)	0.372^c^	0.312	0.416	0.347^c^	0.277	0.429	***
DO (mg/L)	7.86^c^	5.46	10.25	9.55^c^	4.67	21.19	***
pH	7.98^c^	7.51	8.65	7.99^c^	6.30	9.63	
Turb (NTU)	18.26	0.10	115.55	7.64	0.02	82.96	
Density (cells/L)	4.26E6^b^	1.93E5	2.26E8	1.70E7^b^	7.89E4	2.28E8	***
Biomass (mg/L)	0.163^b^	0.012	5.768	1.450^b^	0.006	18.327	***
Shannon	1.032	0.188	1.772	1.207	0.077	2.266	**
Evenness	0.399^a^	0.093	0.756	0.334^a^	0.077	0.961	***

Significance level:

a denotes p<0.05,

b denotes p<0.01,

c denotes p<0.001, blank denotes p >0.05, from Friedman test.

*** denotes p<0.001, ** denotes p<0.01, blank denotes p >0.05, from Kruskal–Wallis H test.

### 3.2 Phytoplankton community and response to environmental factors

Phytoplankton density and biomass varied significantly among the different water layers and were significantly lower in CJ than in XX (**Table 1**). Annual cycles showed two peaks in spring (March~April) and summer (July~August) for both CJ and XX (**Supplementary Figure S1**), and the vertical structure of phytoplankton density and biomass is shown in **Supplementary Figures S2** and **S3**. Diatoms dominated in all layers for both sites. In the first year, the spring peak species were *Stephanodiscus* sp. in CJ and *Stephanodiscus* sp. and *Fragilaria* sp. in XX, while the summer peak in XX was more pronounced with *Stephanodiscus* sp., cyanophytes and cryptophytes. In the second year, density and biomass decreased in CJ, while the summer peak in XX exceeded that of the first year, dominated by *Stephanodiscus* sp., *Synedra acus* and some cyanophytes. Mantel test showed that more environmental factors were significantly correlated with phytoplankton density and biomass in XX compared to CJ, except pH and Turb (**Figure 3**). CJ showed significant relationships only with Temp and DO.

**Figure 4:**
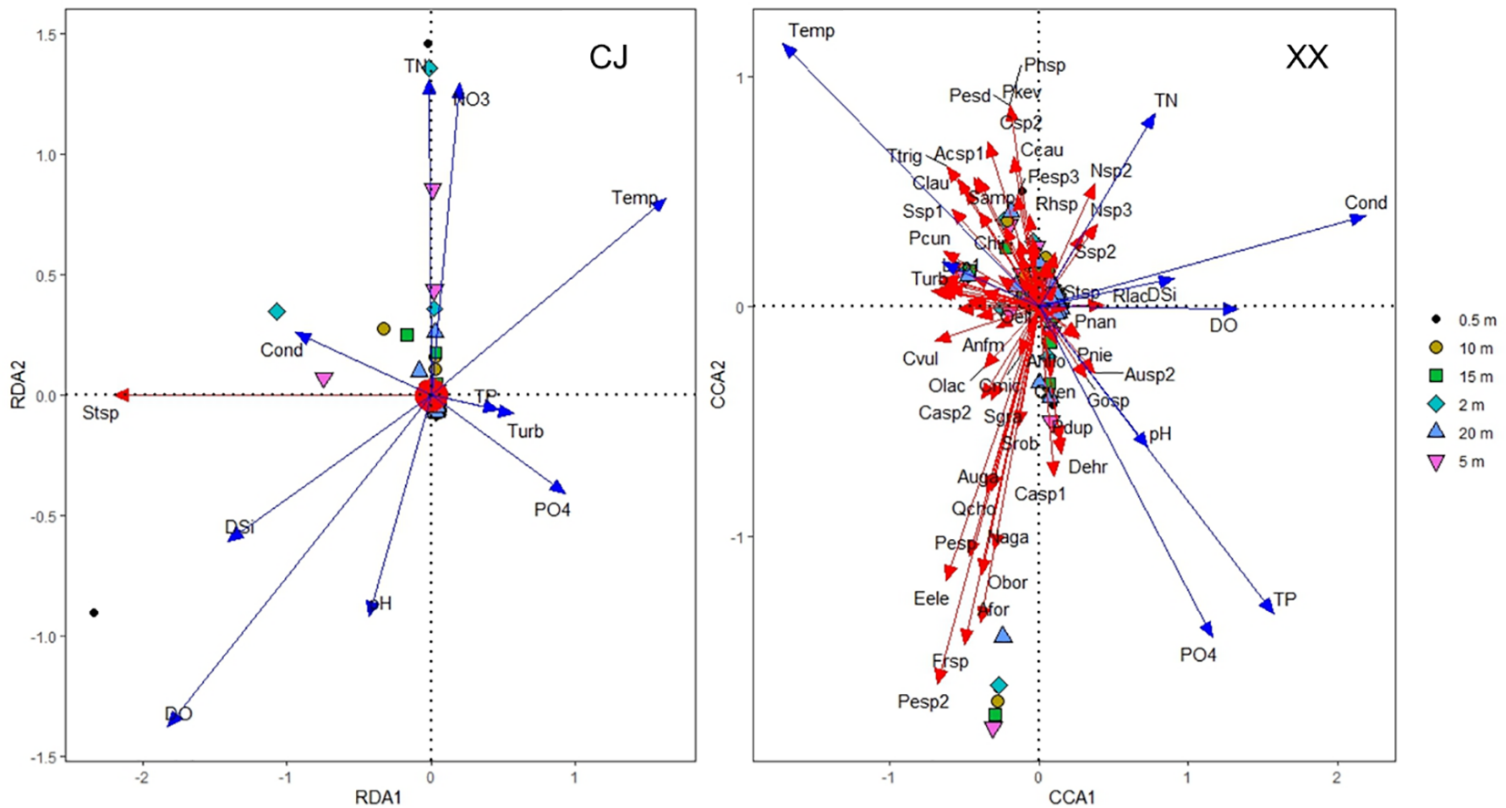
Phytoplankton community ordination based on RDA for CJ and CCA for XX.

RDA and CCA were selected for CJ and XX based on the maximum DCA axis length of 2.12 and 4.55, respectively. **Figure 4** shows different patterns of phytoplankton community ordination between CJ and XX. In CJ, the primary RDA axis explained 21.47% of the variability in phytoplankton data, with significant correlations between phytoplankton species and environmental factors including DO (r = -0.62, p = 0.001) and Temp (r = 0.54, p = 0.003). In XX, the first two CCA axes together accounted for 25.21% of the variability in phytoplankton data (axis 1 = 16.57%, axis 2 = 8.64%), with significant correlations between phytoplankton species and environmental factors including Cond (r = 0.72, p = 0.001), Temp (r = -0.57, p = 0.006), and TP (r = 0.52, p = 0.001) on the first CCA axis. Monte Carlo simulation confirmed the significance of all CCA axes (F = 3.638, p = 0.028 for CJ, and F = 7.796, p = 0.001 for XX, 999 permutations). Notably, *Stephanodiscus* sp. (Stsp) stood out significantly in CJ, while few species were significantly different in XX.

**Figure 5:**
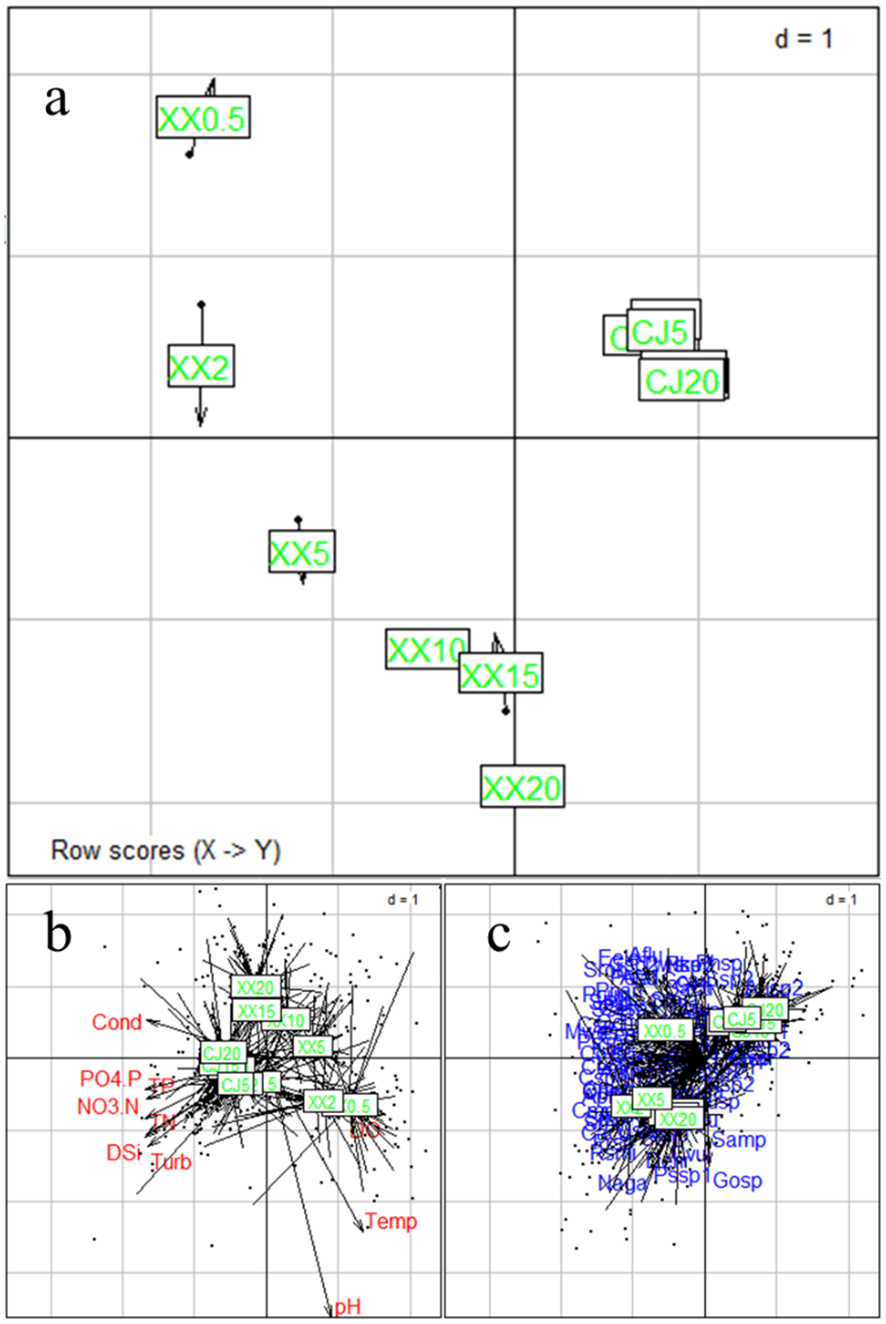
COSTATIS Co-inertia results for sampling locations **(A)**, and intrastructure plot of the COSTATIS method: projection of the sampling locations superimposed with environmental parameters **(B)** and species **(C)**, CJ0.5, CJ2, CJ5, CJ10, CJ15, and CJ20 indicate depths of 0.5 m, 2 m, 5 m, 10 m, 15 m, and 20 m for CJ, and XX0.5, XX2, XX5, XX10, XX15, and XX20 indicate depths of 0.5 m, 2 m, 5 m, 10 m, 15 m, and 20 m for XX.

COSTATIS is used to identify stable structures in phytoplankton species-environment relationships, ignoring temporal structures. The first eigenvalue is much more important than the following ones (97.0% and 2.8% of the total inertia for the first two axes). The COSTATIS co-inertia results for sampling sites are shown in **Figure 5A**, where the tip and the black bullet end of the arrow represent the site from the perspective of phytoplankton and environmental parameters, respectively. The lengths of the arrows are greater in XX than in CJ, indicating a greater discrepancy between phytoplankton and environmental factors. The closer proximity of the points in CJ compared to XX indicates that the water layers in CJ have a higher similarity in phytoplankton species-environment relationships. **Figures 5B and C** illustrates the intrastructure for the sampling sites overlaid with environmental parameters and species. Most of the environmental factors were in the direction of CJ, while DO pointed to XX. These results can be mutually corroborated with **Table 1**. **Figure 5C** indicates that the differentiation of phytoplankton species in different water layers of XX may be mainly due to a significant disparity of 0.5 m.

### 3.3 Phytoplankton diversity patterns and response to hydrodynamic and environmental conditions

**Figure 6:**
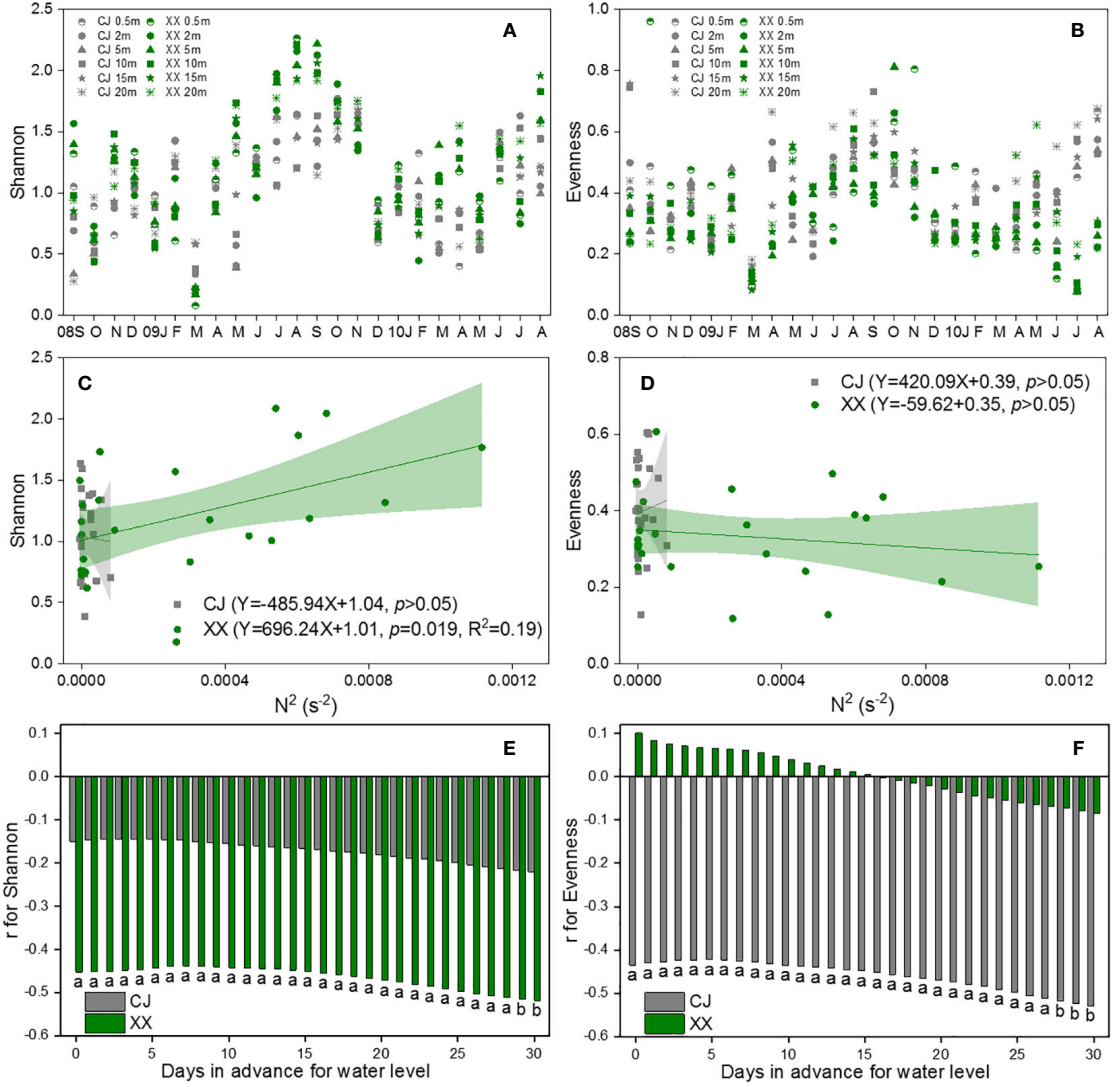
Vertical changes of Shannon **(A)** and Evenness **(B)**, their response to N^2^ **(C, D)** and water level **(E, F)** in CJ and XX. Significance level: ^a^ denotes *p*<0.05, ^b^ denotes *p*<0.01, blank denotes *p >*0.05.

**Figures 6A and B** show the Shannon and Evenness indices of different water layers. Similar temporal cycles were observed for both indices. While the Shannon index showed no significant variation among the water layers, a notable vertical variation was observed for the Evenness index (**Table 1**). Overall, XX had a higher Shannon index while CJ had a higher evenness index (**Figures 6A, B**; **Table 1**). In CJ, significant relationships were observed between the Shannon index and TN, DSi, Temp and DO, while the Evenness index showed significant relationships with DSi, Temp, Cond, DO and Turb (**Figure 3**). However, in XX, the Shannon index only showed significant relationships with Temp, while the evenness index showed significant relationships with NO_3_-N, Temp, and DO.

The Shannon index showed a significant positive relationship with N^2^ in XX, but no relationship with N^2^ in CJ (**Figure 6C**). The evenness index showed no relationship with N^2^ in either CJ or XX (**Figure 6D**). Among the 11 water level indices, the “mean of the daily data before sampling date” index showed significantly more and stronger correlations with the Shannon and Evenness indices. Therefore, it was selected to establish the correlations. The Shannon index showed a negative correlation with water level in XX, with a slight increase observed as the number of days in advance for water level increased (**Figure 6E**). Conversely, the Shannon index did not show a significant relationship with water level in CJ. The evenness index showed a negative correlation with water level in CJ, with a slight improvement observed as the number of days in advance for water level increased (**Figure 6F**). However, the evenness index did not show a significant relationship with water level in XX.

**Figure 7:**
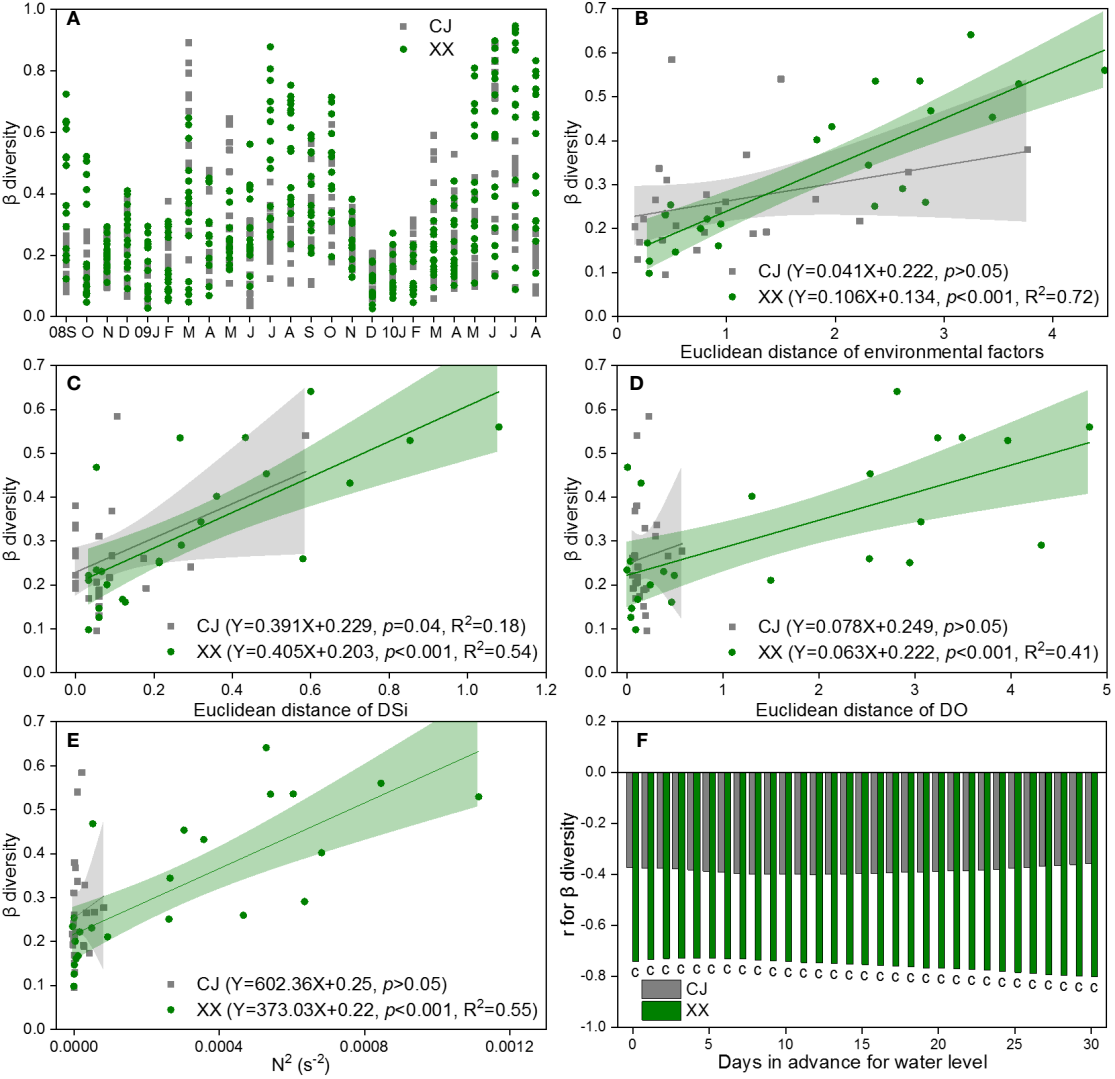
β diversity for pairs of water layers based on Bray-Curtis dissimilarity of community **(A)** and its response to Euclidean distance of environmental factors **(B)**, DSi **(C)** and DO **(D)**, as well as N^2^ **(E)** and water level **(F)** in CJ and XX. Significance level: c denotes *p*<0.001, blank denotes *p >*0.05.

The β diversity for pairs of water layers over time is shown in **Figure 7A**, based on the Bray-Curtis dissimilarity of the community in CJ and XX. In most months, XX had higher β diversity compared to CJ, with exceptions in some spring months. During late autumn and winter, the disparity in pairs of water layers was remarkably low, indicating a uniform distribution of the phytoplankton community in both CJ and XX during these months. DSi (r=0.40, *p*< 0.001) and DO (r=0.39, *p*< 0.001) were identified by Mantel test as the most significant and influential environmental factors related to the differences in community structure between different water layers. The β diversity of the phytoplankton community in XX was found to be significantly positively correlated with the Euclidean distance of the total environmental factor matrix, DSi, and DO (*p*< 0.001, **Figures 7B-D**). However, in CJ, only the difference of DSi in different water layers was significantly positively correlated with β diversity (*p* = 0.04, **Figure 7C**).

The β diversity showed a positive correlation with N^2^ in XX, while no significant relationship with N^2^ was observed in CJ (**Figure 7E**). Among the 11 water level indices, the “mean of the daily data before sampling date” index showed significantly more and stronger correlations with β diversity. Therefore, it was chosen to establish the correlations. The β diversity showed a negative correlation with the water level in XX, and this effect showed a slight increase with an increase in the number of days in advance for the water level (**Figure 7F**). In contrast, no significant relationship was found between β diversity and water level in CJ.

**Figure 8:**
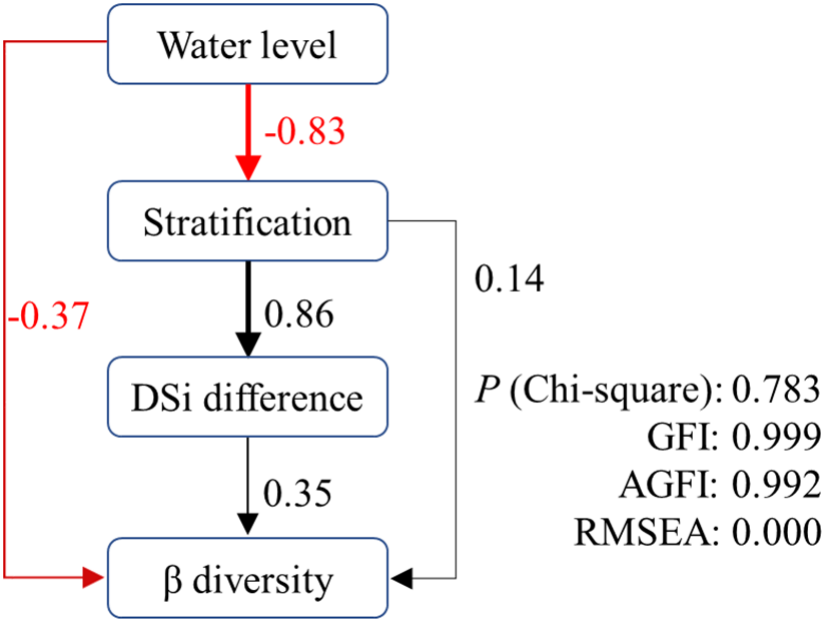
Path with structural equation model predicting direct and indirect effects of water level on β diversity in XX.

Based on the above results, we constructed structural equation models to disentangle the complex interactions of direct and indirect effects of predicted variables on β diversity in XX (**Figure 8**). As hypothesized, both water level and stratification had significant direct and indirect effects on β diversity in XX. Water level had a direct negative effect (-0.37) and stratification had a direct positive effect (0.14) on β diversity. Their indirect effects followed the path of water level → stratification → DSi difference → β diversity. The *p*-value of the Chi-squared test was 0.783, and the goodness-of-fit index (GFI) and adjusted goodness-of-fit index (AGFI) were 0.999 and 0.996, respectively. The root mean square error of approximation (RMSEA) was 0.000. The results indicated that the proposed models provided good interpretations of the original data (Schermelleh-Engel et al., 2003).

## 4 Discussion

### 4.1 Vertical divergences in phytoplankton total density, total biomass and community composition

The aquatic ecosystems in both CJ and XX experienced identical and highly dynamic water level fluctuations during the study period. However, the phytoplankton density in XX was found to be 4 times higher with almost 9 times higher biomass compared to CJ during the two-year study period. This observation highlights the increased risk of algal bloom in the tributary when compared to the mainstream of the reservoir. Kuang et al. (2005) conducted a study on phytoplankton density before the impoundment of TGR and found that phytoplankton density in the tributary was 3.8 times higher than that in the mainstream. After years of operation following the impoundment of TGR, the dynamics of phytoplankton in the tributary become more complicated. The results in **Figure 3** show that phytoplankton density and biomass were only related to Temp and DO in CJ, while almost all the environmental factors influenced phytoplankton density and biomass in XX, supporting the above conclusion to some extent. Although XX had a significantly lower nutrient level as shown in **Table 1**, it had a higher phytoplankton density and biomass. This suggests that nutrient availability did not limit phytoplankton growth in XX.

On the other hand, even in a well-mixed water column, where phytoplankton and nutrients are homogenized throughout the water column, a light gradient is inevitable (Mellard et al., 2011). Therefore, phytoplankton will experience different local light levels and therefore different growth rates. In this study, the turbidity of CJ and XX was relatively low and didn’t show significant differences, but the phytoplankton density and biomass were significantly higher in XX than in CJ and had distinct vertical variations, suggesting that phytoplankton growth is free from light limitation caused by high turbidity in both CJ and XX. This finding is consistent with a study by Liu et al. (2012), and is consistent with the characteristics of the phytoplankton composition. Diatoms are the primary phytoplankton group in the study area and have a low light requirement (Huisman et al., 2004). Both theoretical predictions and field experiments with artificial mixing have shown that diatoms are more efficient species at low light levels than Microcystis (Huisman et al., 2004). Phytoplankton growth was not limited by nutrients or light; therefore, the relatively lower mixing or stronger stratification in XX may facilitate favorable conditions with longer residence time for phytoplankton growth, even though the inflow and outflow of TGR reached their peak for the year during this period. Liu et al. (2012) showed that seasonal thermal stratification is strongly developed in the tributary, but weak in the mainstream of the TGR. Similar findings have been reported in a drinking water reservoir, where thermal intensity (water temperature and thermal stratification intensity) was found to be a key driver of spatiotemporal changes in phytoplankton (Lu et al., 2023).

Throughout the two-year study, phytoplankton density and biomass showed peak values in spring and summer, followed by low values in the autumn and winter, which occurred almost simultaneously for CJ and XX. Significant vertical differences in phytoplankton density and biomass were observed during the peak period, with the surface showing the highest values. A notable finding during this peak period was the significant evolution of phytoplankton from a diatom-dominated community to a chlorophyta/cyanophyta/cryptophyta/dinophyta-dominated community. Turbulence promoted diatom growth, but cyanophyta and dinoflagellates prefer stable water conditions (Rath et al., 2021). A significant disappearance of diatoms was strongly associated with the weakening of mixing. Interestingly, this peak period coincides with the stratification period, which is characterized by relatively high N^2^ values and medium to low water levels. Therefore, both water level and stratification level may influence the vertical differences in total density, total biomass, and the composition of phytoplankton. This conclusion, asserting that the vertical heterogeneity of the phytoplankton corresponds to its vertical hydrological structure, was also confirmed in a 6 m deep lagoon (Radchenko et al., 2023).

When analyzing the responses of phytoplankton community structure to environmental factors, excluding hydrodynamic conditions, it was observed that DO and Temp were significant factors influencing the phytoplankton community in CJ. In contrast, Cond, Temp and TP emerged as significant factors influencing the phytoplankton community in XX. The undeniable effect of temperature on phytoplankton is evident, and in this study, water temperature emerged as a key factor influencing the structure of the phytoplankton community in both CJ and XX. Similar results have been reported in other reservoir systems (Cai et al., 2020; Cui et al., 2023). In addition to water temperature, phytoplankton community structure was more strongly influenced by nutrients in XX compared to CJ. This suggests that nutrients played a more important role in shaping phytoplankton composition in the tributary. While most nutrients showed no vertical variation in CJ, they showed significant differences in the vertical direction in XX, resulting in different phytoplankton compositions in different water layers.

COSTATIS is preferred when species-environment relationships are strong, and temporal structures are not of primary importance (Thioulouse, 2011). In this study, it is applied to test whether stable species-environment relationships can be found in CJ and XX through multiple repeated sampling. The COSTATIS analysis showed that the sites representing the six water layers of CJ were aggregated, while those in XX were quite separated, indicating a more stable species-environment relationship in CJ compared to XX. This means that when considering temporal changes, the species-environment relationship varied significantly in XX, while it remained relatively unchanged in CJ. To some extent, this indicates that CJ has a stronger resistance to external disturbances and the ability to maintain the system structure relatively unchanged. The relatively stable phytoplankton community structure in CJ was consistent with the temporal dynamics of phytoplankton composition in **Supplementary Figures S2** and **S3**. A similar result was reported by Rodrigues et al. (2018), who found high stability of phytoplankton-environment relationships in all reservoir zones except the tributary in a reservoir in central Brazil.

### 4.2 Impact of water level on vertical disparities in phytoplankton diversity

The vertical differences in phytoplankton diversity between CJ and XX are evident in several aspects. As a representative of α diversity, the Shannon index was higher in XX, while the evenness index was higher in CJ, although both showed similar temporal patterns throughout the study period. This suggests that CJ had a more simplified and evenly distributed phytoplankton community structure compared to XX. The significant negative correlation between the evenness index and water level in CJ indicates that, even during the summer with low water levels, the water column in CJ remains relatively mixed, maintaining an evenly distributed phytoplankton community. In contrast, the phytoplankton community in XX exhibited high species diversity during the summer with low water level and strong stratification.

From the perspective of β diversity, CJ showed a much more similar phytoplankton community between different water layers, with a relatively aggregated pattern of low β diversity. The phytoplankton community in CJ maintained a higher level of independence compared to XX, as the β diversity in CJ showed only a weak correlation with DSi. In contrast, in XX, all the environmental factors, including the matrix and single key environmental factors (DSi and DO), as well as N^2^, showed significant and high positive correlations with β diversity in XX. In addition, water level showed a significant and high negative correlation with β diversity in XX. Due to the multitude of factors associated with β diversity in XX, a structural equation model was applied to elucidate the potential pathways through which these factors act. The results showed that both stratification and water level had direct and indirect effects on β diversity in XX, with the direct path involving the DSi difference in water layers.

Water level fluctuation is a complex variable that integrates various physical effects into a comprehensive descriptor (Li et al., 2018). It has been recognized as a key factor influencing phytoplankton biomass and composition in rivers, lakes, and reservoirs (Naselli-Flores and Barone, 1997; Mac Donagh et al., 2009; Wang et al., 2011; Zhu et al., 2013; Yang et al., 2016) through both direct and indirect pathways. Direct effects include biomass dilution and mixing effects during periods of high and low water, respectively. Indirect effects alter the physicochemical characteristics of water bodies, including nutrient variation and underwater light availability (Valdespino-Castillo et al., 2014; Fadel et al., 2015; Liu et al., 2019). These studies have provided valuable insights into how phytoplankton biomass and composition respond to water level effects. However, to the best of our knowledge, the effects of water level on the vertical β diversity of phytoplankton have not been reported.

The reduction of water level during the spring and summer in our study area leads to a more pronounced effect of internal processes with increased residence time. This explains to some extent the direct negative effect of water level on the β diversity of phytoplankton in XX. It also provides a plausible explanation for the occurrence of cyanobacterial blooms following water level drawdowns in certain reservoirs (Cooke, 1980; Yang et al., 2016). The indirect influence of water level acts through stratification and the difference in DSi. The critical role of seasonal stratification in shaping phytoplankton structure and dynamics has been established in reservoir systems (Fonseca and de Mattos Bicudo, 2011; Wang et al., 2011). Our study further confirms that stratification directly affects the variation of community structure throughout the water column, and this stratification is significantly negatively affected by water level (r = -0.83). Stratification promotes an uneven distribution of physiochemical factors in the water column, which in our study significantly increased the DSi difference between water layers (r = 0.86). Silicon is a crucial nutrient for diatom growth, as diatoms need it to build their siliceous cell walls. The high availability of silicon favors the growth of diatoms over non-siliceous phytoplankton (Tréguer and Pondaven, 2000). Consequently, a higher DSi difference induces an increased β diversity of phytoplankton in the vertical direction, thus closing the indirect pathway from water level to β diversity.

To date, numerous researchers have proposed potential strategies for XX under the influence of complex hydrodynamic conditions. Ji et al. (2017) found that the rising water level can lead to an increases or decreases in chlorophyll *a* depending on the water circulation patterns in XX, which were based on both the tributary inflow and the intrusion flow from the TGR. Ye et al. (2022) suggest that the rising water levels have a greater effect on phytoplankton blooms than falling water levels. Gai et al. (2023) suggest a potential dam operation strategy to mitigate blooms during stratification, which involves withdrawing the warm surface water from upstream reservoirs to increase horizontal flows in the surface layer. Through the analysis of vertical phytoplankton data, our study provides a compelling direction for formulating effective water quality management strategies, particularly for XX. Our results highlight the significant negative correlations between β diversity and water level through both direct and indirect effects. During stratification periods characterized by decreased water level, increased phytoplankton density and biomass, along with notable vertical differences of the phytoplankton community, manipulation of water level and application of artificial mixing emerge as promising strategies. The proposed approach involves raising the water level to facilitate dilution and implementing artificial mixing techniques to reduce the β diversity of phytoplankton in different water layers. This approach will not only promote water quality, but also provide a sustainable solution to maintain a balanced ecosystem in the tributary bay. Potential future research directions include: 1) investigating the long-term effects of water level fluctuations on phytoplankton dynamics to understand their ongoing impact on aquatic ecosystems, 2) incorporating advanced modeling techniques to simulate and predict the response of vertical phytoplankton communities to varying environmental conditions to aid in the development of more effective management strategies.

## 5 Conclusion

The study describes the vertical phytoplankton structure and highlights the complex responses of phytoplankton to water level fluctuations and environmental conditions. Monthly data were collected over 2 years in both the mainstream (CJ) and the tributary bay (XX) of the Three Gorges Reservoir. CJ exhibited a more uniform distribution of nutrients across water layers, but maintained a higher overall nutrient level compared to XX. Phytoplankton density and biomass were lower in CJ, which was influenced by water temperature and dissolved oxygen (DO). The phytoplankton community in CJ showed a more stable species-environment relationship, a lower Shannon index and a higher evenness index. This suggests a relatively simple community structure and a more uniform distribution of phytoplankton among different water layers in CJ.

XX had significantly higher phytoplankton density and biomass, influenced by a wide range of environmental factors. The phytoplankton community in XX showed diverse species-environment relationships across different water layers, higher Shannon diversity and a lower evenness. In particular, XX showed increased differences in phytoplankton community between water layers (higher β diversity). These differences showed significant negative correlations with water level and positive correlations with differences in DO, differences in dissolved silica (DSi) and stratification. Peak values of phytoplankton density and biomass, as well as high β diversity in XX, were observed during periods of low water level and strong stratification in spring and summer. A structural equation model complemented by path analysis revealed that a decrease in water level could increase β diversity either directly through internal processes with extended residence time or indirectly by modifying stratification and the vertical distribution of DSi in XX. The substantial disparity in phytoplankton structure characterization between CJ and XX underscores the key role of the tributary in the TGR ecosystem. A proposed water quality management strategy for XX includes raising water levels to facilitate dilution and implementing artificial mixing techniques to reduce the β diversity of phytoplankton across different water layers. The combination of these water quality management methods is expected to effectively control and mitigate algal blooms and help maintain a balanced ecosystem in the tributary bay.

## Data availability statement

The raw data supporting the conclusions of this article will be made available by the authors, without undue reservation.

## Author contributions

LW: Writing – review & editing, Writing – original draft, Visualization, Methodology, Investigation, Formal analysis, Conceptualization. LT: Writing – review & editing, Investigation, Data curation. QC: Writing – review & editing, Supervision, Resources, Project administration, Funding acquisition, Conceptualization.
