# Supplementary material for: Distinct differences of vertical phytoplankton community structure in mainstream and a tributary bay of the Three Gorges Reservoir, China
Source: Front Plant Sci. 2024 Mar 22;15:1381798. doi: 10.3389/fpls.2024.1381798 (PMC10995247; doi:10.3389/fpls.2024.1381798)
Supplement: Supplementary file 1 [file Image_1.pdf]

Supplementary Information

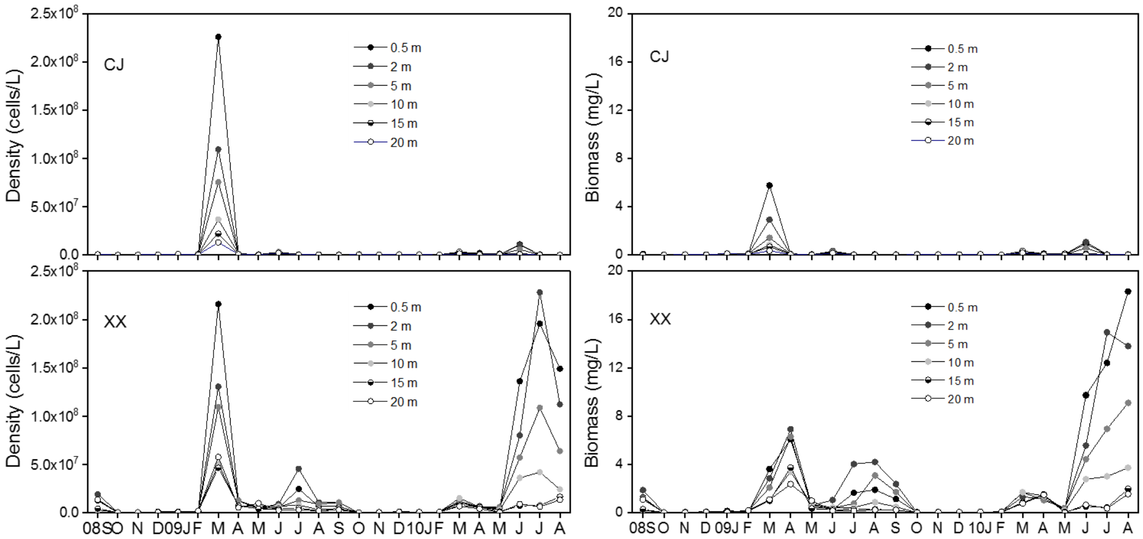

Fig. S1 Vertical density and biomass of phytoplankton in CJ and XX

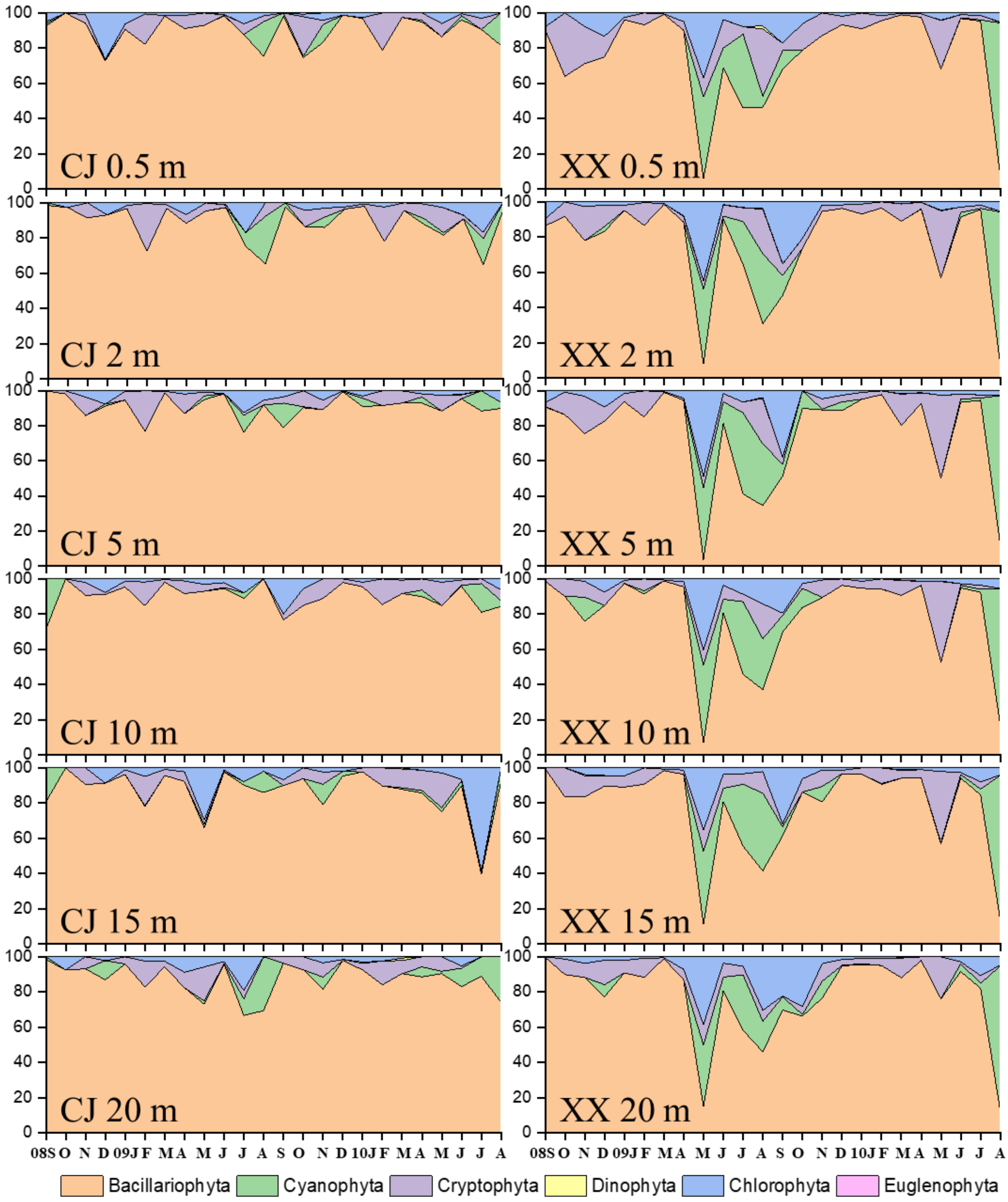

Fig. S2 Density distribution of phytoplankton in different water layers

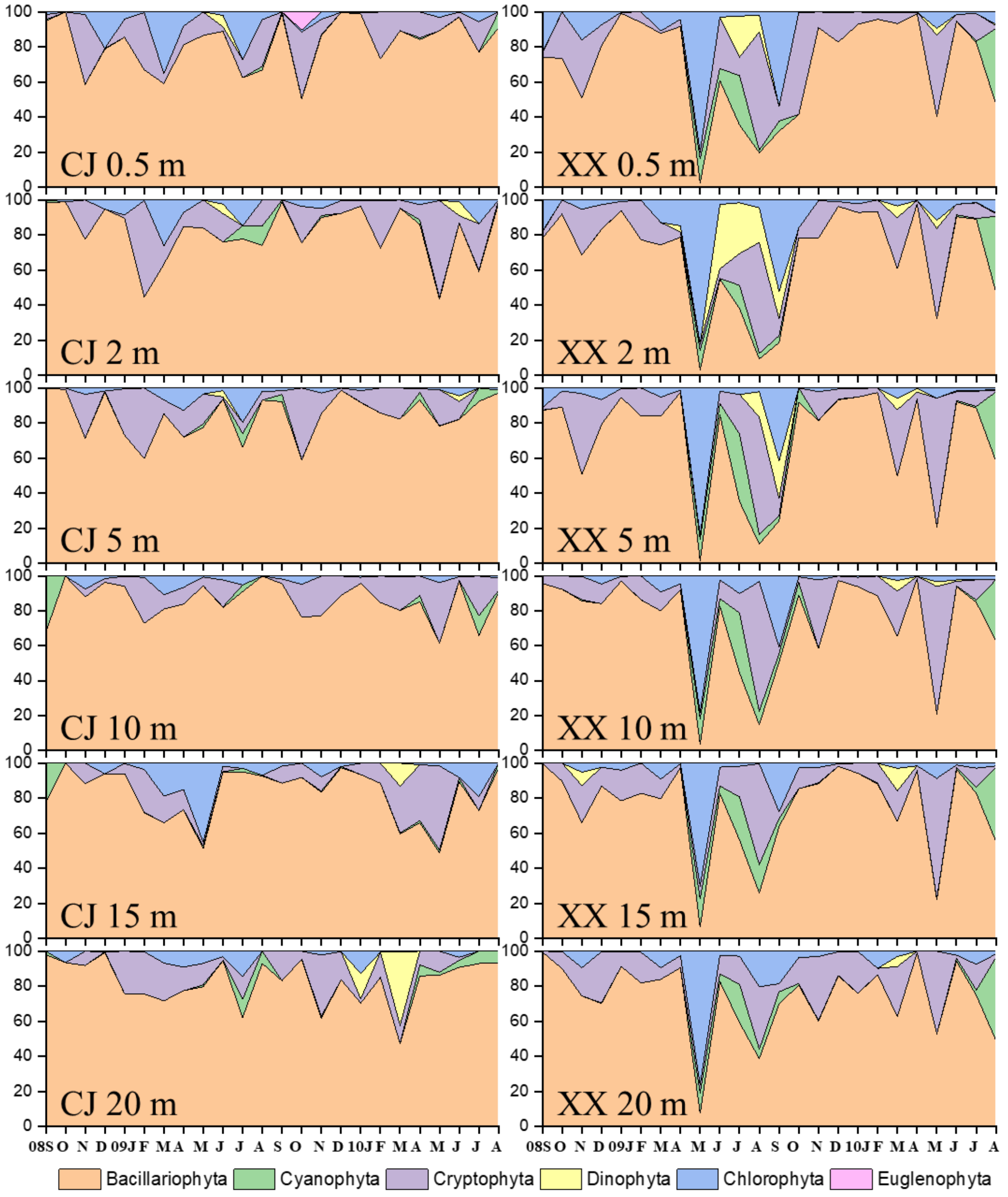

Fig. S3 Biomass distribution of phytoplankton in different water layers
